# Utilization of Point-of-care Echocardiography in Cardiac Arrest: A Cross-sectional Pilot Study

**DOI:** 10.5811/westjem.2021.4.50205

**Published:** 2021-07-20

**Authors:** Yanika Wolfe, YouYou Duanmu, Viveta Lobo, Michael A. Kohn, Kenton L. Anderson

**Affiliations:** * Cooper University Hospital, Department of Medicine, Division of Pulmonary, Allergy & Critical Care, Camden, New Jersey; † Stanford University School of Medicine, Department of Emergency Medicine, Stanford, California

## Abstract

**Introduction:**

Point-of-care (POC) echocardiography (echo) is a useful adjunct in the management of cardiac arrest. However, the practice pattern of POC echo utilization during management of cardiac arrest cases among emergency physicians (EP) is unclear. In this pilot study we aimed to characterize the utilization of POC echo and the potential barriers to its use in the management of cardiac arrest among EPs.

**Methods:**

This was a cross-sectional survey of attending EPs who completed an electronic questionnaire composed of demographic variables (age, gender, year of residency graduation, practice setting, and ultrasound training) and POC echo utilization questions. The first question queried participants regarding frequency of POC echo use during the management of cardiac arrest. Branching logic then presented participants with a series of subsequent questions regarding utilization and barriers to use based on their responses.

**Results:**

A total of 155 EPs participated in the survey, with a median age of 39 years (interquartile range 31–67). Regarding POC echo utilization, participants responded that they always (66%), sometimes (30%), or never (4.5%) use POC echo during cardiac arrest cases. Among participants who never use POC echo, 86% reported a lack of training, competency, or credentialing as a barrier to use. Among participants who either never or sometimes use POC echo, the leading barrier to use (58%) reported was a need for improved competency. Utilization was not different among participants of different age groups (P = 0.229) or different residency graduation dates (P = 0.229). POC echo utilization was higher among participants who received ultrasound training during residency (P = 0.006) or had completed ultrasound fellowship training (P <0.001) but did not differ by gender (P = 0.232), or practice setting (0.231).

**Conclusion:**

Only a small minority of EPs never use point-of-care echocardiography during the management of cardiac arrest. Lack of training, competency, or credentialing is reported as the leading barrier to use among those who do not use POC echo during cardiac arrest cases. Participants who do not always use ultrasound are less likely to have received ultrasound training during residency.

## INTRODUCTION

Point-of-care echocardiography (POC echo) is established as a useful adjunct in the management of cardiac arrest patients in the emergency department (ED) setting, yet there are currently no large studies describing the optimal utilization of the technology in these cases. There are a number of reports describing a potential prognostic role for POC echo, and it has long been promoted as a method to identify the potential etiology of cardiac arrest as well.^1^–^6^ Recent literature has discovered that POC echo is frequently useful in verifying the presence of cardiac activity in cases of pulseless electrical activity and asystole where the management may be substantially altered from traditional Advanced Cardiac Life Support guidelines.^4^,^5^,^7^,^8^ Additionally, POC transesophageal echo in humans has confirmed the finding, first discovered in interventional animal studies, that the traditional location of chest compressions is frequently preventing cardiac output by occluding the aortic outflow tract with each compression.^8^–^10^ Consequently, a few small series have described how POC echo can be used to monitor and correct the quality and location of chest compressions.^5^,^11^–^14^

Despite the recognized utility of POC echo, emergency physicians (EP) do not universally use this imaging modality during cardiac arrest cases, nor do EPs always use POC echo for the same purposes during cardiac arrest management. Since cardiac arrest is one of the leading causes of death for patients over the age of 40, even small improvements in cardiac arrest management with POC echo could potentially save thousands of lives annually.^15^ Although larger trials are needed to understand how POC echo can be used to optimize cardiac arrest management, we also need to begin to explore EP utilization of this technology.

In this pilot study, we aimed to better characterize the utilization and the potential barriers to the use of POC echo during cardiac arrest among EPs. This pilot will be used to validate survey questions, and the response variability will be used to calculate sample size for future larger-scale studies.

## METHODS

### Study Design and Setting

We developed. an anonymous, cross-sectional survey in Research Electronic Data Capture (REDCap, Vanderbilt University, Nashville, TN), and a link to the survey was emailed to a convenience sample of attending EPs at six academic and two community EDs in five different states (CA, NV, WA, PA, MA) over a two-month period in 2019 (Appendix A). Two reminder e-mails were sent in an effort to increase response rate; no incentives were provided. Study sites were intentionally chosen on the East and West Coasts of the United States to increase generalizability of the results but were limited to locations where the study investigators had contacts who they believed would contribute to a meaningful response rate to the survey. The study was approved by the institutional review board with a waiver of documentation of informed consent; reporting follows the STROBE statement for observational studies.^16^

### Intervention

The survey questions were developed by four point-of-care ultrasound (POCUS) fellowship-trained EPs and were tested on an additional 20 EPs with mixed prior ultrasound experience to validate that the survey items were clear, understandable, and relevant to the construct. The survey that ultimately was sent to the study population was the third iteration, which had undergone minor changes to increase clarity or to decrease the potential for incorrect entries based on these initial survey responses. The survey consisted of five demographic questions including level of prior ultrasound training and practice location, as well as four questions regarding the utilization of POC echo during cardiac arrest (Appendix A).

If participants answered “always” or “sometimes” to question 1, “How often do you use point-of-care echocardiography during cardiac arrest cases?” then branching logic in the REDCap^T^ survey presented questions 2–4 regarding the use of POC echo during cardiac arrest. If participants answered “never” to question 1, then questions 5 and 6 were presented rather than questions 2–4. Question 6 was also presented to participants who answered “sometimes” to question 1.

### Outcomes

The purpose of this pilot study was to assess for trends in both the utilization and the perceived barriers to the use of POC echo during cardiac arrest in the emergency department (ED) setting. POC utilization (always vs sometimes and never) was compared with the demographic variables listed in Table 1. Additionally, we compared the proportion of the first half of respondents who answered “always” to the proportion from the second half of respondents to determine whether there was a temporal relationship to the responses. Participants also had the option to write in answers to echo utilization and barrier questions by choosing “other” options to allow additional insight into these variables at this pilot stage of the survey (Appendix B).

### Analysis

We calculated and reported descriptive statistics with 95% confidence intervals (CI). The relationship between respondent demographics and POC echo utilization was assessed using prevalence ratios with 95% CIs, and *P*-values calculated using Fisher’s exact test. We used chi-squared analysis to compare early survey participants and late participants regarding frequency of POC echo utilization. We performed analyses using Stata15.1/SE for Windows (StataCorp, LP, College Station, TX).

## RESULTS

A total of 155 EPs participated in the survey, with a median age of 39 years (interquartile range 31–67). The total survey response rate was 56% (95% CI, 50, 62); the response rate for academic centers was 52% [95% CI, 45, 58] and for community centers was 90% (95% CI, 80, 100). Demographic information is provided in Table 1. The majority of participants graduated from residency in the last decade, had received ultrasound training during their residency, and practiced in an academic setting.

A small minority of participants reported that they never use POC echo during cardiac arrest cases; all seven of the non-users were from the academic group, and most of those reported lack of training, competency, or credentialing as a barrier (Table 1). Among participants reporting that they either sometimes or never used POC echo during cardiac arrest, they cited a need for improved competency as the leading barrier to use (Table 1). There was no temporal difference in the proportion of participants who reported always using POC echo; 51 (77%) of the first half of participants and 51 (78%) of the second half of participants reported always using POC echo during cardiac arrest (*p* = 0.91).

Utilization of POC echo was not different among participants of different age groups or participants with different residency graduation dates (Table 2). Regarding the remaining demographic variables, POC echo use was higher among participants who received ultrasound training during residency or had received ultrasound fellowship training. Only two (29%, 95% CI, 8.2, 64) of the seven non-users graduated from residency prior to 2000, and four (57%, 95% CI, 25, 84) had not received any ultrasound training in residency.

## DISCUSSION

In this pilot study, we found that participants who do not use POC echo during cardiac arrest reported a lack of training, competency, or credentialing as the leading barriers to utilization. Participants who do not always use ultrasound are less likely to have received ultrasound training during residency. Our findings are consistent with prior studies which have reported that common barriers to POC ultrasound utilization, in general, are lack of training, departmental flow requirements, and lack of access to an ultrasound machine.^17^–^20^ In this study, one noteworthy finding is that neither age nor residency graduation date resulted in less POC echo use.

Point-of-care ultrasound training in the ED began during the 1990s with the introduction of the Focused Assessment with Sonography in Trauma (FAST) exam to the United States and steadily increased until the Accreditation Council for Graduate Medical Education (ACGME) designated ultrasound as one of the 23 milestone competencies for emergency medicine (EM) residency graduates in 2012.^21^ While it is unlikely that any of the study participants who graduated before 1990 received ultrasound training during residency, POCUS training has been mandatory for all EM residents since 2012. Consequently, it would seem that age, graduation date, and ultrasound training would have similar, inter-related results in regard to POC echo utilization, but that is not the case in this pilot study. This result is due, in part, to older participants who have learned to use ultrasound after residency graduation; in fact, the three oldest participants reported that they always use POC echo.

There is also variability in the quality of POCUS training among residency training programs. Some participants who graduated since 2012 and responded that they did not receive POCUS training, may have answered that way because they either did not receive high quality POCUS training or they may not have had enough training in the use of POC echo during cardiac arrest to feel comfortable with its use during resuscitation. This finding suggests that there may be educational deficiencies preventing the use of POC echo, and that more work is needed to determine what these deficiencies in training are and how they can be overcome. Of note, recent literature suggests that general POC echo knowledge during cardiopulmonary resuscitation may not be sufficient since the use of POC echo can decrease compression ratio during cardiac arrest; an understanding of how to minimize echo time during chest compression pauses, or institutional protocols for the same purpose, may be necessary; the use of transesophageal echocardiography (TEE) rather than transthoracic echocardiography (TTE) also improves compression ratio since there is no time spent during pauses searching for an adequate cardiac window.^22^–^26^

In this pilot study, we also found no difference in POC use between academic and community settings. The number of community physicians that participated was much smaller than the number of academic physicians; so this trend may not persist in a larger study. There may be more perceived barriers to POC echo use in the community setting (eg, fewer personnel for assistance); thus, if future work does find that this trend persists, it may suggest that EPs who are adequately trained in either setting understand the importance of using POC echo during cardiac arrest and will incorporate it into their practice despite the presence of perceived barriers. Remarkably, all seven participants who responded that they never used POC echo were from academic centers; it is likely that a larger community sample would have non-users as well.

The purpose of this pilot study was to provide the first-ever characterization of the utilization and the potential barriers to the use of POC echo during cardiac arrest among EPs. To this end the study has fulfilled its purpose. However, as a pilot study this investigation had an inherently small sample size, especially among community EPs, and should not be misconstrued as being widely generalizable. Our group is currently planning a larger study using national organization listservs to recruit a more representative sample of both academic and community EPs nationwide. This pilot has also provided some external validity to the survey tool. The free-text answers (Appendix B) suggest that the survey questions were well understood overall, but they elucidated some minor changes that can still be made to provide further clarity. In addition, the response variability from this pilot will be used to calculate sample size in future studies. We hope that this further work will be valuable in identifying barriers to POC echo utilization and will help guide interventions to overcome those barriers.

## LIMITATIONS

The primary limitations of this study were the small sample size and the response rate. Although small, the sample size was appropriate for this pilot study; our primary aim was to characterize the use of POC echo among a convenience sample of EPs. Although our response rate was higher than anticipated, it still leaves room for non-response bias. It is possible that participants in this study were more experienced with POC echo and were thus more interested or more comfortable in answering a survey on the topic; this may be particularly true of the community ED group where all of our participants reported using POC echo at least some of the time during cardiac arrest. However, there was no temporal difference in how participants responded to the question regarding frequency of POC echo use; this finding suggests that non-response bias may have been minimized since participants who required multiple reminders before responding submitted similar responses to early participants.

## CONCLUSION

In our pilot survey of emergency physicians, only a small minority never use point-of-care echocardiography during cardiac arrest in clinical practice. Lack of training, competency, or credentialing was reported as the leading barriers to utilization among those who do not use POC echo during cardiac arrest cases. Participants who do not always use ultrasound are less likely to have received ultrasound training during residency.

## Supplementary Information





## Figures and Tables

**Table 1:** Demographic data and answers to the echocardiography utilization questionnaire.

Variable	N	% (95% CI)
Gender		
Male	88	57 (49–65)
Female	67	43 (35–51)
Age, years		
30–39	80	52 (44–59)
40–49	56	36 (29–43)
50–59	9	5.8 (2.1–9.4)
60–69	8	5.2 (1.7–8.6)
70–79	2	1.2 (0.3–4.5)
Residency graduation year		
2010–2018	94	61 (53–68)
2000–2009	43	28 (21–35)
1990–1999	10	6.4 (2.6–10)
1980–1989	5	3.2 (0.4–6.0)
1970–1979	2	1.3 (0.5–3.0)
Ultrasound training in residency	134	86 (81–92)
Ultrasound fellowship training	25	16 (10–22)
Practice setting		
Academic	133	86 (80–91)
Community	22	14 (8.7–20)
Pediatric only*	8	5.2 (1.6–8.6)
1. How often do you use point-of-care echocardiography during cardiac arrest cases? Choose one:		
Always	102	66 (58–73)
Sometimes	46	30 (22–37)
Never	7	4.5 (1.2–7.8)
2. What type of point-of-care echocardiography do you use during cardiac arrest cases? Choose one:		
TTE	147	99 (98–100)
TEE	0	0.0 (0.0–0.0)
Both	1	1.0 (0.6–2.0)
3. When do you use point-of-care echocardiography during cardiac arrest cases? Choose all that apply:		
Beginning of resuscitation	69	47 (39–55)
End of resuscitation	116	78 (72–85)
During pulse/rhythm checks	124	84 (78–90)
Other**	9	6.1 (2.2–9.9)
4. What do you use point-of-care echocardiography for during cardiac arrest cases? Choose all that apply		
To identify potentially treatable causes	132	89 (84–94)
To prognosticate	141	95 (92–99)
To evaluate chest compression quality	4	2.7 (0.1–5.3)
Other**	1	0.7 (0.1–3.7)
5. Why don’t you use point-of-care echocardiography during cardiac arrest cases? Choose all that apply:		
Lack of ultrasound training, competency, or credentialing	6	86 (42–100)
Lack of support from literature or national recommendations	1	14 (2.5–51)
Limited ultrasound machine availability	1	14 (2.5–51)
Technical challenges	1	14 (2.5–51)
Liability for incorrect use	1	14 (2.5–51)
“Other**	1	14 (2.5–51)
6. Which of the following would make it more likely for you to use point-of-care echocardiography during cardiac arrest cases? Choose all that apply:		
Improved competency	31	58 (45–72)
Credentialing status	8	15 (5.4–25)
Known survival benefits	21	40 (26–53)
More accessible ultrasound machines	13	25 (13–36)
More physical space	14	26 (15–38)
An assistant	25	47 (34–61)
Other**	1	1.9 (0.3–9.9)

* All were academic.

** See Appendix 2 for free-text answers to “Other.”

*CI*, confidence interval; *TTE*, transthoracic echocardiography; *TEE*, transesophageal echocardiography.

**Table 2:** Point-of-care echocardiography utilization compared to demographic variables.

Demographic variable	Always N (%)	Sometimes or Never N (%)	Prevalence ratio	95% CI	*P*-value
Age					
30–39 years (n=80)	58 (73)	22 (28)	REF		
40–49 years (n=56)	31 (55)	25 (45)	0.76	0.58–1.0	0.045
50+ years (n=19)	13 (68)	6 (11)	0.94	0.68–1.3	0.779
Residency Graduation Year					
2010–2018 (n=94)	65 (69)	29 (31)	REF		
2000–2009 (n=43)	25 (58)	18 (42)	0.84	0.63–1.12	0.246
1970–1999 (n=17)	11 (65)	6 (35)	0.94	0.64–1.36	0.779
Gender					
Male (n=88)	54 (61)	34 (39)	REF		
Female (n=67)	48 (72)	19 (28)	1.2	0.93–1.5	0.232
Ultrasound Training					
No (n=21)	8 (38)	13 (62)	REF		
Yes (n=134)	94 (70)	40 (30)	1.8	1.1–3.2	0.006*
Ultrasound Fellowship Training					
No (n=130)	78 (60)	52 (40)	REF		
Yes (n=25)	24 (96)	1 (4)	1.6	1.4–1.9	<0.001*
Practice Setting					
Community (n=22)	17 (77)	5 (23)	REF		
Academic (n=126)	79 (63)	47 (38)	1.2	0.9–1.6	0.231

* significant difference, p<0.05

*CI*, confidence interval; *REF*, reference group.
