# Supplementary material for: Utilization of Point-of-care Echocardiography in Cardiac Arrest: A Cross-sectional Pilot Study
Source: West J Emerg Med. 2021 Jul 20;22(4):803–9. doi: 10.5811/westjem.2021.4.50205 (PMC8328182; doi:10.5811/westjem.2021.4.50205)
Supplement: Supplementary file 1 [file wjem-22-803-s001.pdf]

Appendix A. Survey

Consent

This survey is part of a research project explained in the attached information sheet. By completing and submitting this survey, you affirm that you are at least 18 years old and that you give your consent for the investigators to use your answers in their research.
[Attachment: "Utilization of Point-of-Care Echo in Arrest Information Sheet.pdf"]

Demographic Information

What is your sex?
○ Male
○ Female

What is your age (years)? __________

What year did you graduate from residency? [▼]

Did you receive any point-of-care ultrasound training during your residency?
Choose one:
○ Yes
○ No

Have you completed ultrasound fellowship training?
Choose one:
○ Yes
○ No

Where are you currently practicing? [▼]

In your practice, do you only take care of pediatric patients only?
○ Yes
○ No

Echocardiography Survey

1. How often do you use point of care echocardiography during cardiac arrest cases?
Choose one answer:
○ Always
○ Sometimes *
○ Never **

2. What type of point-of-care echocardiography do you use during cardiac arrest cases?
Choose one answer:
○ Transthoracic Echocardiography
○ Transesophageal Echocardiography
○ Both

3. When do you use point-of-care echocardiography during cardiac arrest cases?
Choose all that apply:
☐ At the beginning of the resuscitation
☐ At the end of the resuscitation
☐ During pulse/rhythm checks
☐ Other
Please Specify: __________

4. What do you use point-of-care echocardiography for during cardiac arrest cases?
Choose all that apply:
☐ To identify potentially treatable causes (such as cardiac tamponade, hypovolemia, proximal aortic dissection, right heart strain, etc...)
☐ To prognosticate through evaluation for cardiac activity (organized activity, cardiac standstill, VF, etc...)
This includes, but is not limited to, evaluation just prior to terminating resuscitative efforts
☐ To evaluate chest compression quality.
☐ Other
Please Specify: __________

5. Why don't you use point-of-care echocardiography during cardiac arrest cases?
Choose all that apply:
☐ Lack of ultrasound training, competency, or credentialing
☐ Lack of support from literature or national recommendations
☐ Limited ultrasound machine availability
☐ Technical challenges (e.g. time consuming, physical space limitations, patient factors
☐ Liability for incorrect use
☐ Other
Please Specify: __________

6. Which of the following would make it more likely for you to use point-of-care echocardiography during cardiac arrest cases?
Choose all that apply:
☐ Improved competency
☐ Credentialing status
☐ Known survival benefits
☐ More accessible ultrasound machines
☐ More physical space
☐ An assistant (someone else to perform the point-of-care echocardiography so I can focus on other nuances of the resuscitation)
☐ Other
Please Specify: __________

** Participants that answered 'Never' were provided with additional questions 5 and 6.
** Participants that answered 'Never' were not provided with question 2-4.
* Participants that answered 'Sometimes' were provided with additional question 6.
