# Supplementary material for: Utilization of Point-of-care Echocardiography in Cardiac Arrest: A Cross-sectional Pilot Study
Source: West J Emerg Med. 2021 Jul 20;22(4):803–9. doi: 10.5811/westjem.2021.4.50205 (PMC8328182; doi:10.5811/westjem.2021.4.50205)
Supplement: Supplementary file 2 [file wjem-22-803-s002.pdf]

Appendix B. Free text answers to 'Other' from Table 1.

**3. When do you use point-of-care echocardiography during cardiac arrest cases?**
If any question of tamponade.
Intermittently.
US guided procedures. During CPR (fast, Aorta, etc).
Usually try to get a cardiac view sometime early on, but I don't allow it to interrupt chest compressions, and then again at the end or towards the end.
Whenever I suspect or question absence of cardiac activity.
Depends on many variables. Compressions take priority, but identifying tamponade/complete standstill influences decisions.
To work through a differential of reversible causes during the resuscitation.
During pulse check, I quickly take a clip of the fem artery/vein and then look it online, as the code proceeds.
To rule out reversible causes if cause initially unknown.
**4. What do you use point-of-care echocardiography for during cardiac arrest cases?**
Just would like to point out that if we had a TEE probe I would use it for compression quality and continuously during resuscitation.
**5. Why don't you use point-of-care echocardiography during cardiac arrest cases?**
It's hard enough to get basics achieved (ABC's) with limited equipment without introducing more equipment.
**6. Which of the following would make it more likely for you to use point-of-care echocardiography during cardiac arrest cases?**
We have a basic supply issue (i.e. airway, lines, etc). Ultrasound just complicates things.
